# Dosimetric evaluation of synthetic CT for magnetic resonance-only based radiotherapy planning of lung cancer

**DOI:** 10.1186/s13014-017-0845-5

**Published:** 2017-06-26

**Authors:** Hesheng Wang, Hersh Chandarana, Kai Tobias Block, Thomas Vahle, Matthias Fenchel, Indra J. Das

**Affiliations:** 1 Department of Radiation Oncology, New York University School of Medicine, Langone Medical Center, New York, NY USA; 2 Bernard and Irene Schwartz Center for Biomedical Imaging, Department of Radiology, New York University School of Medicine, New York, NY USA; 3 Siemens Healthcare GmbH, Erlangen, Germany

**Keywords:** MR-only radiotherapy, Synthetic CT, Radiotherapy treatment planning, Lung cancer

## Abstract

**Background:**

Interest in MR-only treatment planning for radiation therapy is growing rapidly with the emergence of integrated MRI/linear accelerator technology. The purpose of this study was to evaluate the feasibility of using synthetic CT images generated from conventional Dixon-based MRI scans for radiation treatment planning of lung cancer.

**Methods:**

Eleven patients who underwent whole-body PET/MR imaging following a PET/CT exam were randomly selected from an ongoing prospective IRB-approved study. Attenuation maps derived from the Dixon MR Images and atlas-based method was used to create CT data (synCT). Treatment planning for radiation treatment of lung cancer was optimized on the synCT and subsequently copied to the registered CT (planCT) for dose calculation. Planning target volumes (PTVs) with three sizes and four different locations in the lung were planned for irradiation. The dose-volume metrics comparison and 3D gamma analysis were performed to assess agreement between the synCT and CT calculated dose distributions.

**Results:**

Mean differences between PTV doses on synCT and CT across all the plans were −0.1% ± 0.4%, 0.1% ± 0.5%, and 0.4% ± 0.5% for D95, D98 and D100, respectively. Difference in dose between the two datasets for organs at risk (OARs) had average differences of −0.14 ± 0.07 Gy, 0.0% ± 0.1%, and −0.1% ± 0.2% for maximum spinal cord, lung V20, and heart V40 respectively. In patient groups based on tumor size and location, no significant differences were observed in the PTV and OARs dose-volume metrics (*p* > 0.05), except for the maximum spinal-cord dose when the target volumes were located at the lung apex (*p* = 0.001). Gamma analysis revealed a pass rate of 99.3% ± 1.1% for 2%/2 mm (dose difference/distance to agreement) acceptance criteria in every plan.

**Conclusions:**

The synCT generated from Dixon-based MRI allows for dose calculation of comparable accuracy to the standard CT for lung cancer treatment planning. The dosimetric agreement between synCT and CT calculated doses warrants further development of a MR-only workflow for radiotherapy of lung cancer.

## Introduction

Lung cancer comprises nearly 33% of all cancers with relatively high mortality rate in every ethnic group and gender [1]. Radiation therapy is the main treatment option for lung cancer. The 5-year overall survival rate after radiotherapy in patients with stage 1 non-small cell lung cancer is nearly 50%. When patients are properly selected and treated using hypo-fractionated stereotactic body radiation therapy (SBRT), the survival rate increases to 90-95% depending on the size of tumor and dose [2].

MR images have been routinely used for contouring tumors and organs at risks (OARs) in radiotherapy due to the excellent soft tissue contrast. Common practice is to register the MR images to planning CT, and then to transfer MR-contoured structures to the planCT for treatment planning. This process can potentially introduce systematic errors due to uncertainties in the MR-to-CT registration. Moreover, dedicated CT simulation scans add radiation dose, additional cost, inconvenience to patient and examination time for the patients. Therefore, the interest to implement a MR-only treatment planning workflow has grown rapidly, especially in view of the recent emergence of MRI-linear accelerator technology [3, 4].

Use of MRI data for treatment planning has been limited by two main challenges with MR images: i) geometric distortions [5] and ii) lack of electron density information. Various studies have shown that improved scanner design, advanced MR sequences and correction schemes could reduce spatial distortion in MR images to acceptable level for radiation therapy [4, 6, 7]. Electron density information is required for accurate dose calculation to account for the tissue heterogeneity. The lung exhibits substantial tissue inhomogeneity. Algorithms with inhomogeneity correction provides superior (<2%) dose distribution [8] and changes clinical practice and prescription dose in lung SBRT due to its refinement of dose calculation [9, 10]. Modern treatment planning derives electron density data from CT simulation [11–14]. To enable MR-based treatment planning, the technique called synthetic CT (synCT), a pseudo CT derived from MR images, is gaining momentum for treatment planning and dose evaluation in modern days where inhomogeneity correction is universally applied.

Various uses of synCT in treatment planning have been reported [15–22]. However, clinical use especially in the lung is very limited. The aim of this study is to investigate the feasibility of using a MR-generated synCT for treatment planning of lung cancer where the impact of inhomogeneity is expected to be significant, and to evaluate the dosimetric differences between synCT and conventional CT-planned doses.

## Methods

### Image acquisition

Whole-body CT from PET/CT exams and subsequent MR imaging of 11 patients (mean age ± standard deviation (SD): 50 ± 17 years old; range: 25–75 y) were included in this study. These patients included 3 with bone metastases, 2 with breast mass, 1 with lung cancer, 1 with peritoneal implant, and 4 with no lesions. All patients were part of an institutional review board (IRB)-approved clinical study and provided written informed consent. In the study, patients first underwent a clinically indicated PET/CT examination and then a whole-body PET/MRI acquisition. The CT was acquired according to a standard clinical protocol on a PET/CT scanner (Biograph mCT, Siemens AG Healthcare). The CT acquisition used a setting of 100–140 kVp and had a pixel size of 1.52 × 1.52 mm^2^ or 1.37 × 1.37 mm^2^ with a slice thickness of 5.0 mm. The MR data was obtained on a 3 T whole-body MR/PET hybrid system (Biograph mMR, Siemens Healthcare) using a 2-point, 3-dimensional volume-interpolated breath-holding Dixon sequence [23]. The sequence used the following parameters: 3.6 ms repetition time (TR), 2.46 ms echo time (TE), 10° flip angle and 19 s acquisition time for MR images with 192 × 126 pixel in-plane dimension and 128 slices in coronal orientation (voxel size: 2.60 × 2.60 mm^2^, slice thickness: 3.12 mm).

### Attenuation map generation

A previously published method [24] that generates linear attenuation coefficients (LACs) at 511 keV photon (attenuation map) for PET attenuation correction was adapted to generate synCT from the Dixon MR scans. The method consists of two steps: 1) segmentation of soft tissue on the MR images using an automatic classification method [25], and 2) addition of bone structures to the soft-tissue attenuation map using a model-based bone segmentation algorithm. The soft-tissue segmentation approach classified Dixon MR volumes into 4 tissue types: air, fat, lung, and soft tissue, and then assigned LACs of 0.0 cm^−1^, 0.0854 cm^−1^, 0.0224 cm^−1^ and 0.1 cm^−1^ for the tissue types, respectively [24].

The model-based bone segmentation was described in detail in the previous publication [24]. In brief, to add bone information, a model that included a set of pre-aligned MR images and bone mask pairs was constructed offline. The bone masks contain the LACs of each major body bone, including left and right upper femur, left and right hip, spine and skull. The MR images in the model were registered to the patient’s MR images using a two-stage registration [24]. Subsequently, using the same spatial transformation, the bone masks were registered to the patient image space, which then enables adding the bone structures to the patient’s attenuation map. Depending on bone masks, LACs for bone were between 0.1 and 0.2485 cm^−1^ [24].

### Image preparation

To prepare the data for treatment planning, the MR-generated attenuation maps for 511 keV photon were first converted into CT images (i.e., synCT) assuming a conventional 100-140kVp x-ray source that is commonly used in standard CT scanners. The transformation was performed by using the standard bilinear conversion to get CT Hounsfield unit (HU) of −1000, −110, −767 and 70 for air, fat, lung and soft tissue, respectively [26]. Bone obtained from the model-based segmentation had HU between 70 and 2661. Subsequently, the coronal synCT and planCT were reformatted into axial orientation. Both images were then interpolated to have a voxel size of 1.25 × 1.25 × 2.5 mm^3^ that is typically used in our clinic for lung cancer treatment. At the end, the synCT were registered to planCT by using first rigid and then deformable registrations in Velocity (VelocityAI 3.1.0, Velocity Medical Solutions, Altanta, GA). The registration focused on the body part from the mandible to superior aspect of the kidneys to optimize the alignment of the lung and related bony structures. We also cropped the whole body synCT and planCT superiorly and inferiorly to the region required for treatment planning of lung cancer. Alignment between synCT and CT was evaluated visually. Furthermore, mean HUs in relevant organs were calculated and compared in HU numbers to assess the intensity correspondences between the two images.

### Treatment planning

Both synCT and planCT were imported into the treatment planning software Eclipse (Eclipse 11.0.47; Varian Medical Systems, Palo Alto, CA). For dose calculation, Eclipse transferred the HU into an electron density using a calibration curve obtained from a simulation CT scanner in our radiation oncology department. OARs including heart, spinal cord, and left/right lung were manually contoured on the synCT. A spherical planning target volume (PTV) was created inside the lung to simulate a lung lesion for radiation treatment. As the artificial PTV was in the lung, the air density of the PTV was overridden as water (HU −4) to represent tumor density. Finally, an IMRT plan was designed on the synCT to treat the PTV using 5, 7, or 9 beams. The plan was optimized using the conventional dose objectives for lung cancer treatment and normalized to have 95% PTV covered by 100% prescribed dose. The dose prescription was either IMRT dose of 59.40 Gy in 1.8 Gy/fraction for 33 fractions or SBRT dose of 60 Gy in 20 Gy/fraction for 3 fractions. The PTV with overridden density, OARs, and plan were copied to the planCT. The plan was then recalculated on the planCT using the same MUs as the synCT plan. Dose calculations on both synCT and planCT used the analytic anisotropic algorithm (AAA_11031, Eclipse) for heterogeneity correction with a calculation grid size of 2.5 × 2.5 mm^2^.

### Plan evaluation

To evaluate the effects of PTV size and location on synCT-based treatment planning, we created 18 small-sized PTVs (vol: 11.9 ± 5.5 cm^3^), 13 medium-sized PTVs (vol: 83.5 ± 14.5 cm^3^), and 12 large-sized PTVs (vol: 222.3 ± 39.8 cm^3^) randomly on the 11 patients. Of these PTVs, 9 were located at the lung apex, 16 were near the mediastinum, 9 were at the lung periphery near ribs, and the other 9 were at the posterior periphery close to spine. Treatment planning, dose calculation, and the comparison as described below were performed for each of the target volumes.

Dose distributions between synCT and planCT were compared using the global 3D gamma analysis [27] at criteria of 1%/1 mm, 2%/2 mm and 3%/3 mm (dose difference/distance to agreement) with dose threshold of 10%. Voxels with gamma index smaller than 1 were the points where the synCT dose agreed with the CT dose based on given acceptance criteria. Dose-volume metrics were evaluated for the contoured OARs and PTVs from their dose volume histograms (DVHs). The dose in the lung (excluding PTV) was evaluated using V10 and V20, and heart dose was compared by V40, where Vx is the percentage volume of the OAR receiving dose larger than x Gy. The spinal-cord dose was compared by maximal dose in the structure. The PTV D95, D98 and D100 were calculated to compare the dose in a target volume, where Dx is the percentage dose covering x% PTV.

### Statistics

Paired *t*-test with significance level of 0.05 was performed in all the tumors to test the relative agreement of the dose-volume metrics between synCT and planCT-calculated doses. In addition, the nonparametric Wilcoxon *U*-test was performed on the differences of the metrics between groups based on tumor size and location to evaluate whether the differences related to tumor size or location. Both tests were performed using Matlab R2012b (The MathWorks, Natick, MA).

## Results

### Synthetic CT

Mean HUs in OARs were measured on synCT and planCT for each of the eleven patients (Table 1). T-tests indicated that synCT has comparable mean HUs as conventional CT in the lung and soft tissue (all *p* > 0.05). The HUs in the bone and spinal cord had statistically significant differences between the synCT and planCT. Comparing the standard errors of the HUs, synCT exhibited less inter-patient variations, consistent with the fact that the synthetic HUs were assigned according to tissue classification from the MR images.

**Table 1:** Average HU numbers of OARs in synCT and planCT

Pt#	R Lung		L Lung		Spinal Cord		Heart		Spine Bone	
	synCT	CT	synCT	CT	synCT	CT	synCT	CT	synCT	CT
Pt1	−747	−637	−740	−616	72	33	68	120	268	244
Pt2	−752	−701	−737	−693	72	69	70	38	252	349
Pt3	−747	−774	−738	−766	72	55	68	130	260	422
Pt4	−730	−745	−701	−702	62	64	70	45	260	299
Pt5	−692	−714	−698	−721	67	49	70	49	260	365
Pt6	−714	−739	−690	−726	74	78	69	44	266	505
Pt7	−730	−650	−685	−674	109	52	66	41	269	300
Pt8	−740	−741	−727	−706	72	70	70	42	260	405
Pt9	−734	−812	−734	−793	65	42	68	31	256	249
Pt10	−759	−775	−758	−775	74	46	65	40	266	292
Pt11	−760	−838	−760	−822	86	57	63	146	268	268
Mean ± SE	−737 ± 6	−739 ± 19	−724 ± 8	−727 ± 18	75 ± 4	56 ± 4	68 ± 1	66 ± 13	262 ± 2	336 ± 25
*P*-value	0.90		0.88		0.007		0.90		0.014	

### Dosimetric comparison

**Fig. 1:**
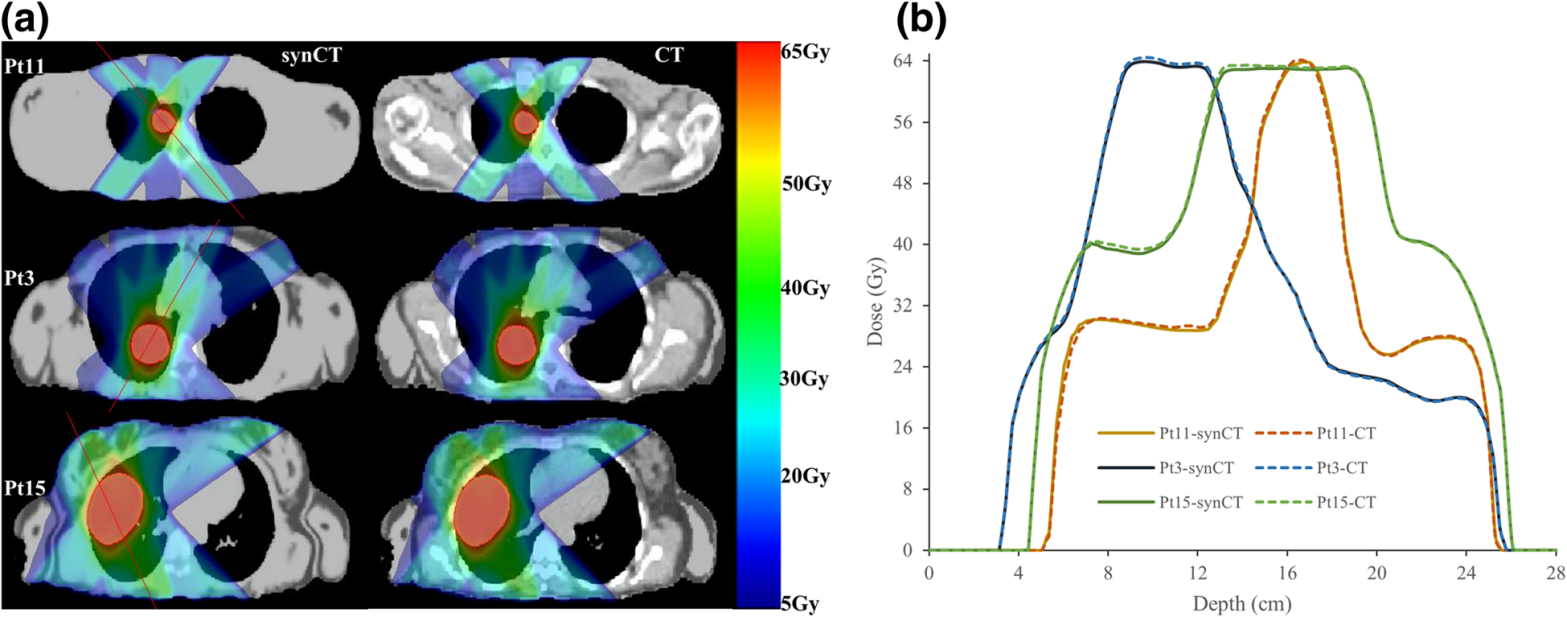
(**a**): MR synthetic CTs (*left*) and standard planCTs (*right*) overlapped with planned doses for a small tumor of Pt11 (*top row*), medium-sized tumor of Pt3 (*middle row*) and large-sized tumor of Pt15 (*bottom row*). PTVs were contoured; a red line across the tumor was drawn for dose line analysis. (**b**): doses along the drawn lines for the three patients

**Fig. 2:**
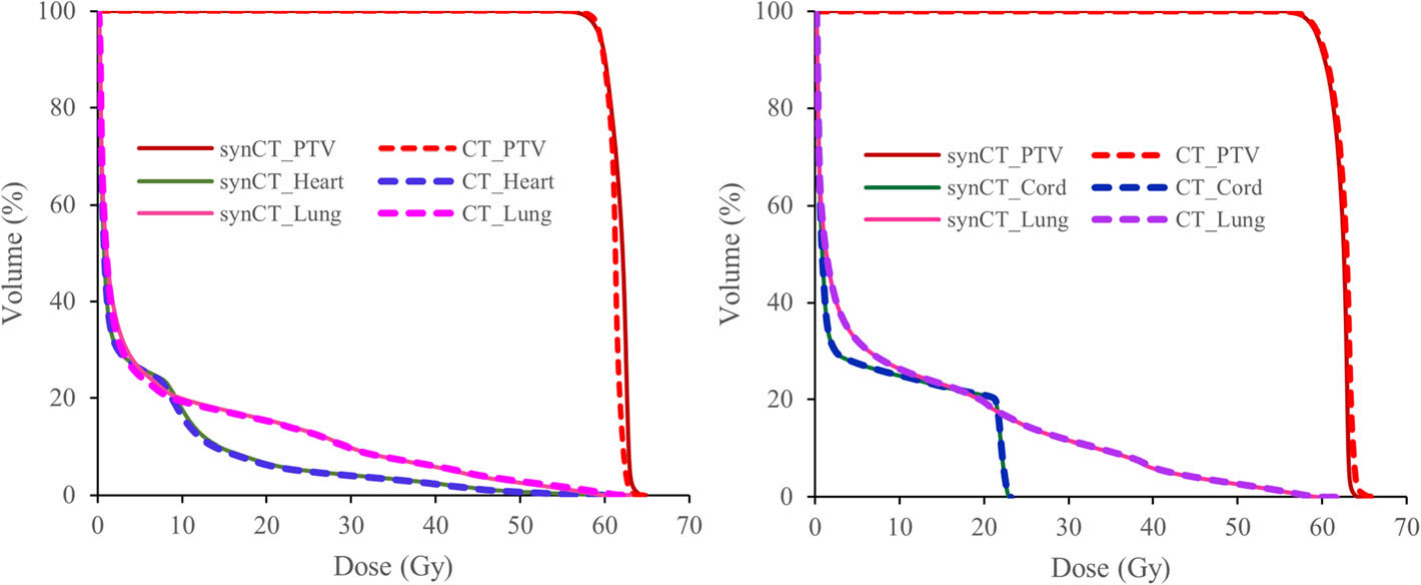
The DVH curves for the OARs and PTV for Pt#11 (*left*) and Pt#15 (*right*)

**Fig. 3:**
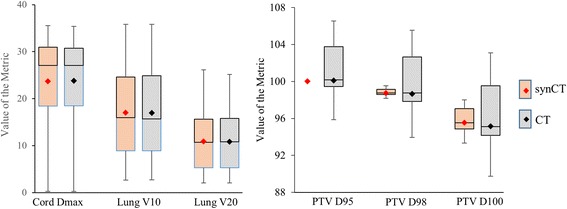
Dose-volume metrics in all the plans for OARs (*left*) and PTV (*right*)

Figure 1 shows planed dose distributions overlapped on synCT and planCT for three patients with different tumor locations and sizes. The solid and dashed dose lines for synCT and CT on the right panel demonstrate very good agreement. Sample DVHs for a medium-sized and a large-sized tumor are shown in Figure 2. The PTV and OARs dose-volume metrics were extracted from the curves for each plan of the total 43 target volumes. In all plans, the mean differences in spinal cord max dose, lung V10 Gy, lung V20 Gy, and heart V40 Gy between synCT and CT were −0.14 ± 0.07 Gy (mean ± 95% confidence interval (CI)), 0.0% ± 0.1%, 0.0% ± 0.1% and −0.1% ± 0.2%, respectively. Figure 3a show a whisker plot comparing mean, median, 25%, and 75% of the metrics between two images. It shows no significant differences in lung V10 (*p* = 0.66) and V20 (*p* = 0.47), or heart V40 (*p* = 0.25) between the two images. Mean heart V40s were 2.8% and 2.7% for synCT and CT, respectively. Maximum dose in the spinal cord exhibited a significant difference (*p* < *0.01*), while the difference ranged from −0.57 to 0.56 Gy. The insignificant and small differences in PTV D95, D98, and D100 from synCT to planCT suggest that the synCT and planCT datasets provide near identical dosimetric data.

Table 2 shows mean differences between the dose-volume metrics subdivided according to tumor size and location. No significant differences in the lung or heart metrics were observed in any tumor group (*p > 0.2*). However, the spinal cord maximum dose presented a statistically significant difference when the tumors were located at lung apex (*p* = 0.001), likely due to the minor misregistration effects between synCT and CT at the neck region. The differences in PTV metrics were also insignificant (*p* > 0.06) between synCT and CT within any group.

**Table 2:** Mean differences and 95% confident intervals of dosimetric metrics between synCT and planCT calculated doses

		small-sized	medium-sized	large-sized	Near Spine	Near Trachea	Near Ribs	Lung Apex
Lung	V10 (%)	0.0 ± 0.0	0.0 ± 0.2	−0.1 ± 0.0	0.0 ± 0.1	0.0 ± 0.1	0.0 ± 0.0	0.0 ± 0.1
	V20 (%)	0.0 ± 0.1	−0.1 ± 0.1	0.1 ± 0.0	0.1 ± 0.2	0.0 ± 0.1	0.0 ± 0.0	0.0 ± 0.1
Heart	V40 (%)	0.0 ± 0.0	−0.1 ± 0.2	−0.1 ± 0.0	−0.2 ± 0.3	0.0 ± 0.1	0.0 ± 0.0	0.0 ± 0.0
Spinal Cord	Dmax (Gy)	−0.2 ± 0.1	−0.1 ± 0.2	−0.1 ± 0.1	−0.2 ± 0.2	−0.1 ± 0.1	−0.1 ± 0.1	−0.3 ± 0.1
PTV	D95 (%)	0.1 ± 0.9	−0.2 ± 0.5	−0.3 ± 0.0	−0.7 ± 0.8	−0.2 ± 0.7	1.0 ± 0.0	−0.5 ± 0.6
	D98 (%)	0.3 ± 1.0	−0.2 ± 0.5	−0.1 ± 0.0	−0.5 ± 1.0	0.0 ± 0.7	1.2 ± 0.0	−0.3 ± 0.7
	D100 (%)	0.7 ± 1.1	0.0 ± 0.6	0.2 ± 0.0	−0.1 ± 1.3	0.3 ± 0.8	1.3 ± 0.0	0.0 ± 0.7

Furthermore, the Wilcoxon *U*-test was used to evaluate whether the dose-volume metric differences between synCT and planCT depended on tumor size or location. No significant differences were observed in any OAR metric between tumors with different sizes (*p *> 0.2) or locations (*p* > 0.05). PTV dose metrics changes from synCT to planCT also exhibited insignificant differences for any two tumor-size groups (*p* > 0.3). But the increase of PTV D95 (1.0%) and D98 (1.2%) at synCT for the tumors near ribs were statistically different from those changes for tumors at any of the other locations (*p* ≤ 0.03).

### Gamma analysis

**Fig. 4:**
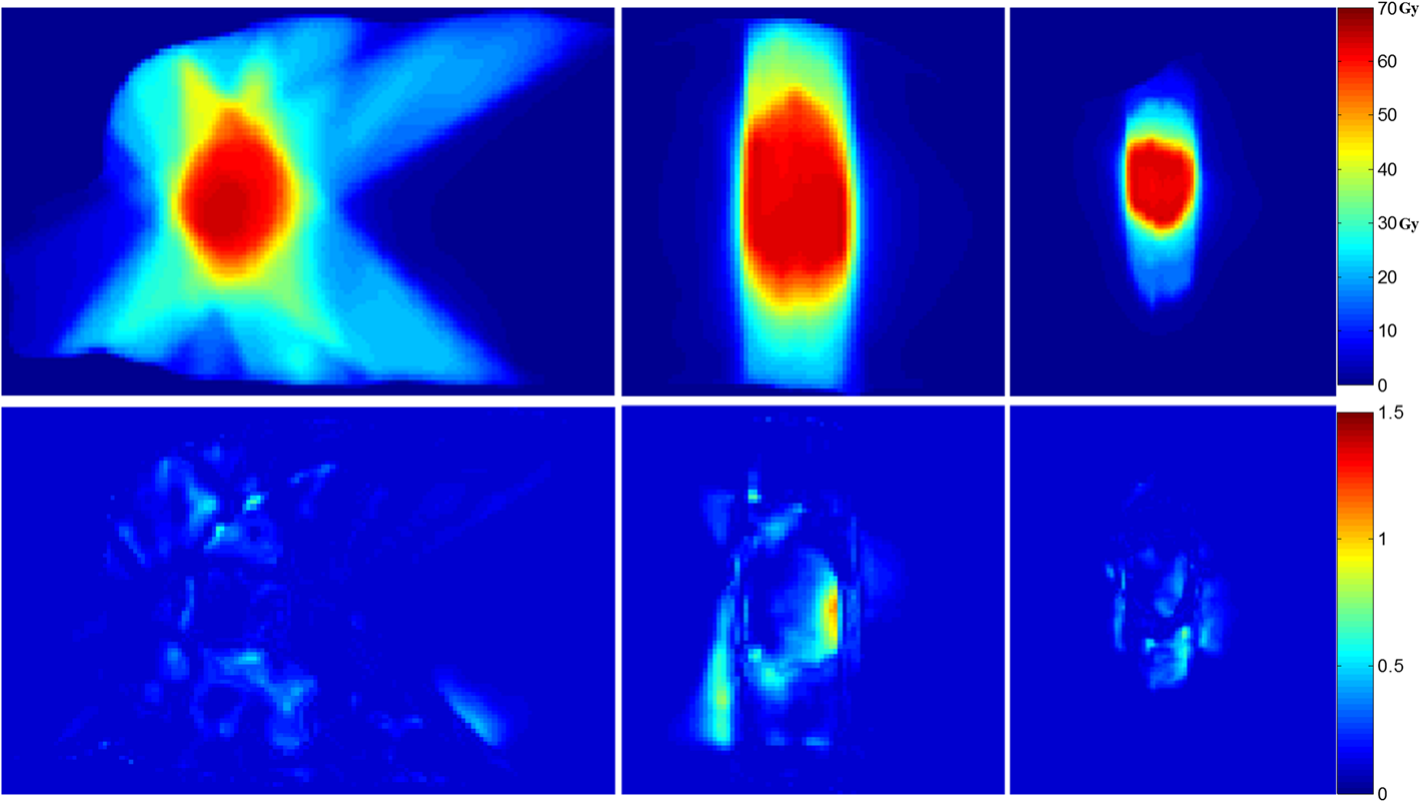
Sample dose distributions from a synCT plan (*top row*) versus 2%/2 mm 3D gamma maps (*bottom row*) at an axial (*first column*), coronal (*middle column*) and sagittal (*right column*) slice

Figure 4 lower panel shows a sample 2%/2 mm gamma map in three orthogonal planes through the isocenter for pt#15, revealing that 100% points on synCT and planCT-calculated doses agreed with each other. Regardless of tumor size and location, the mean passing rate of gamma analysis between synCT and CT calculated doses (Table 3) is greater than 99% at a clinically-used 2%/2 mm criterion. At 1%/1 mm criterion, tumor at lung apex showed higher passing rate than the tumors at other locations.

**Table 3:** Means and standard deviations of gamma analysis

	Nvol	1%/1 mm	2%/2 mm	3%/3 mm
small-sized	18	94.6 ± 4.6	99.2 ± 0.9	99.9 ± 0.2
medium-sized	13	95.1 ± 3.1	99.4 ± 0.7	99.9 ± 0.1
large-sized	12	95.5 ± 5.6	99.3 ± 1.8	99.9 ± 0.2
Near Spine	9	93.8 ± 4.8	99.2 ± 0.8	99.9 ± 0.1
Near Trachea	16	94.6 ± 4.3	99.2 ± 0.9	99.9 ± 0.2
Near Ribs	9	94.0 ± 5.3	99.0 ± 1.9	99.9 ± 0.2
Lung Apex	9	98.0 ± 1.5	99.8 ± 0.3	100.0 ± 0.1
Total	43	95.0 ± 4.4	99.3 ± 1.1	99.9 ± 0.1

*NVol* Number of PTV volumes

## Discussion

This study evaluated the feasibility of pseudo CT images generated from MR scans for treatment planning of lung cancer where dosimetric differences are expected to be high due to tissue inhomogeneity. The treatment plans were optimized on synCT and recalculated on planCT. The synCT plans provided identical dose distribution, without cases in which the plans calculated on planCT exceeded OAR dose constraints or underdose in target volumes. The dose-volume metrics and 3D gamma analysis showed that dose distribution calculated on synCT sufficiently agreed with that on conventional planCT. The agreement suggests that MR-derived synCT can be utilized in the treatment planning workflow to support MR-only radiotherapy of lung cancer with inhomogeneity correction [9, 10].

Using MR-generated synCT for treatment planning has been investigated in RT of cancer in the brain, head and neck, prostate, and pelvis [15, 17, 20, 22], where target volumes were within bulky tissues. In this study, we showed that synCT can generate equivalent treatment plans as standard CT for radiotherapy of cancer in the lung. Regardless of the size and location of a tumor, the synCT plans agreed with CT dose distribution with 99.3% passing rate using a 2%/2 mm acceptance criterion in gamma analysis. Unlike previous studies that used 2D gamma analysis on an image slice [17, 18, 20], we used 3D analysis to evaluate entire irradiated volume. Although matching closely, the mean dose in PTV between synCT and planCT calculations showed a mean difference of −1.2% ± 0.3% with *p* < 0.001. The density inhomogeneity from lung to tumor may increase the sensitivity of the dose calculation to HU variation in the lung due to photon attenuation and electron transport [9]. Gamma analysis with 1%/1 mm criterion showed that tumors at lung apex had a higher pass rate than the tumors at other locations, maybe due to the fact that the tumors at lung apex had less air-tissue inhomogeneity.

A common strategy to generate synthetic CT from MR is to segment or classify MR volumes into various tissue types based on tissue characteristics in MR and subsequently assign HUs to the tissue types [15, 18]. However, it is typically challenging to separate bone from air as both present as signal void in conventional Dixon MR images. Ultrashort echo time (UTE) images improve contrast between air and bone, but are susceptible to misclassification of cartilage and tendon as bone [16]. Furthermore, UTE scans can be limited in obtaining high-quality images in a large volume of interest [28] and may prolong scan time. In this study, we used a model-based method that derived bone information by matching patient MR images with an offline-constructed bone model, which does not require an additional MR scan. This approach has been previously used for PET attenuation correction in whole-body PET/MRI imaging, especially in bony tissue and nearby soft tissue [24]. This study suggests that the method potentially supports a hybrid PET/MRI radiotherapy simulator by providing a tool for both PET attenuation correction and generation of treatment planning synCT.

MR-only treatment planning avoids the systematic uncertainty in MR-to-CT registration for conventional CT-based planning. But an atlas-based method to generate synCT from MR need a registration between patient MR and atlas images [29], which may be prone to registration errors like the MR-to-CT registration. The method we used is a hybrid method in which only bone information is derived from the atlas registration, while soft tissue is directly classified from patient MR images. As the atlas is used only for bone segmentation, a specially-designed two-stage registration method is used [24], which consisted of a landmark-based registration and then an intensity-based deformable registration. The initial bone landmark-based registration is likely to improve the deformable registration. Therefore, this method may improve bone segmentation compared with classification-based method, with minimal uncertainty in the atlas registration.

The group average HUs in organs on synCT matched well to the standard HUs on conventional CT. The synCT assigned a constant HU to each tissue type, leading to smaller HU variations across patients as indicated in Table 1. In comparison, CT exhibited intensity variations specific to the individual patient. Fortunately, doses of high-energy photons are relatively insensitive to small local electron density variations [8]. In addition to typically-used tissue types of air, fat, muscle, and bone [15, 20], the method used here included lung as tissue type for the dose calculation. In this method, we only accounted for major osseous structures such as spine, and the ribs were not included in the atlas. However, dosimetric evaluation of the ribs is necessary for irradiation of lung tumors abutting to the ribs, especially in SBRT. Incorporating ribs into the bone model may improve dosimetric agreement of synCT plans with CT plans in tumors neighboring the ribs. As shown in Table 2, compared with other groups, the tumors near the ribs showed significantly higher PTV D95 and D98 with synCT compared to planCT.

To minimize the effect of geometrical discrepancy between MR and CT on dosimetric evaluation, CT and MR scans should be ideally aligned. In this study, MR was obtained from a breath-hold scan, while CT data was acquired was under free breathing. We therefore performed a nonrigid registration between synCT and CT before treatment planning. However, the challenge to register piecewise-rigid vertebrate bones accurately, especially in the neck region, may lead to significant differences in spinal cord maximum dose when tumors are located at the lung apex, although the differences seen in this study were within the range from −0.51Gy to 0.08Gy. To reduce this effect, a possible option is to employ a free-breathing MRI technique such as the Radial-VIBE sequence, which has shown improved quality compared to conventional VIBE sequence in abdominal imaging [30]. In addition, geometrical distortions induced by tissue susceptibility at air-tissue interfaces should be assessed and corrected if needed [31, 32]. The susceptibility effects increase with magnetic field strength, but can somewhat be alleviated by increasing the readout bandwidth.

Currently, PET/CT is the standard imaging modality for target volume contouring in radiotherapy of lung cancer. Use of MRI in evaluation of lung nodules has been limited due to the susceptibility effects near air-tissue interfaces and the presence of respiratory and cardiac motion artifacts in the images. However, the development of high-performance gradient system, parallel imaging, triggered acquisition and optimizing imaging sequences has made it possible to use MR images for contouring target tumors and organs at risk in radiotherapy [33, 34]. The 4D MRI has shown potential to delineate moving target volumes and assess the range of tissue motion in three-dimensions [35, 36].

A limitation of this study is that the CT and MR images were acquired in an IRB-approved clinical study on diagnostic radiology scanners rather than on dedicated CT-simulator or MR scanners in a radiation oncology department. Recently, dedicated MR simulator has been employed in several radiation oncology departments [37, 38]. The MR systems have a 60–70 cm wide bore to accommodate most cancer patients. The scanners have flat table top and MR-compatible immobilization devices to image a patient in the same position as in radiation treatment. The MR surface coil is mounted on holders over the patient, so its anterior part does not contact with patient body and its posterior part is inside or below the table top. We are currently evaluating the role of these imaging techniques directly in our radiotherapy-simulation and treatment-planning workflow. Nevertheless, this study demonstrates the potential value of MR-generated synCT for radiotherapy planning of lung cancer. Further evaluation of MR-based treatment planning for lung cancer is warranted.

## Conclusions

A method to generate attenuation maps from a set of standard whole-body MR images has been adapted and evaluated for generating synCT for treatment planning in radiation treatment of lung cancer. The plans calculated on synCT agreed closely with the dose computed on standard planCT. This study warrants further evaluation of MR-based treatment planning for lung cancer in a larger patient cohort.

## Abbreviations

CT: Computed tomography
DVH: Dose volume histogram
HU: Hounsfield Unit
LAC: Linear attenuation coefficients
MR: Magnetic Resonance
OAR: Organ at risk
PlanCT: Planning CT
PTV: Planning target volume
SBRT: Stereotactic body radiation therapy
SynCT: Synthetic CT
